# Thoracic Epidural Anesthesia Can Be Effective for the Short‐Term Management of Ventricular Tachycardia Storm

**DOI:** 10.1161/JAHA.117.007080

**Published:** 2017-10-27

**Authors:** Duc H. Do, Jason Bradfield, Olujimi A. Ajijola, Marmar Vaseghi, John Le, Siamak Rahman, Aman Mahajan, Akihiko Nogami, Noel G. Boyle, Kalyanam Shivkumar

**Affiliations:** ^1^ UCLA Cardiac Arrhythmia Center and Neurocardiology Center of Excellence UCLA Health System David Geffen School of Medicine at UCLA Los Angeles CA; ^2^ UCLA Department of Anesthesiology UCLA Health System David Geffen School of Medicine at UCLA Los Angeles CA; ^3^ University of Tsukuba Tsukuba Japan

**Keywords:** autonomic nervous system, electrical storm, thoracic epidural anesthesia, ventricular tachycardia storm, Arrhythmias, Autonomic Nervous System

## Abstract

**Background:**

Novel therapies aimed at modulating the autonomic nervous system, including thoracic epidural anesthesia (TEA), have been shown in small case series to be beneficial in treating medically refractory ventricular tachycardia (VT) storm. However, it is not clear when these options should be considered. We reviewed a multicenter experience with TEA in the management of VT storm to determine its optimal therapeutic use.

**Methods and Results:**

Data for 11 patients in whom TEA was instituted for VT storm between July 2005 and March 2016 were reviewed to determine the clinical characteristics, outcomes, and role in management. The clinical presentation was incessant VT in 7 (64%), with polymorphic VT in 3 (27%) and monomorphic VT in 8 (73%). The underlying conditions were nonischemic cardiomyopathy in 5 (45%), ischemic cardiomyopathy in 3 (27%), and hypertrophic cardiomyopathy, Brugada syndrome, and cardiac lipoma in 1 (9%) each. Five (45%) had a complete and 1 (9%) had a partial response to TEA; 4 of the complete responders had incessant VT. All 4 patients with a documented response to deep sedation demonstrated a complete response to TEA.

**Conclusions:**

More than half of the patients with VT storm in our series responded to TEA. TEA may be effective and should be considered as a therapeutic option in patients with VT storm, especially incessant VT, who are refractory to initial management. Improvement in VT burden with deep sedation may suggest that sympathoexcitation plays a key role in perpetuating VT and predict a positive response to TEA.

**Clinical Perspective**

**What Is New?**

- In patients who were seen with ventricular tachycardia storm, refractory to medical management and initiation catheter ablation, for which thoracic epidural anesthesia was administered, more than half had complete or >80% reduction in ventricular tachycardia episodes; a documented response in ventricular tachycardia burden to deep sedation appears to predict a positive response to thoracic epidural anesthesia.

**What Are the Clinical Implications?**

- Thoracic epidural anesthesia should be considered in patients with ventricular tachycardia refractory to initial medical and/or ablation therapy and who do not have absolute contraindications to epidural catheter placement while awaiting definitive therapy.

The autonomic nervous system, particularly the sympathetic nervous system, plays an important role in the pathogenesis of ventricular tachycardia (VT) storm.^1^, ^2^, ^3^ Neuraxial modulation shows promise in the management of VT storm. Successful treatment of VT storm with cardiac sympathetic blockade has been reported with surgical cardiac sympathetic denervation (CSD),^4^, ^5^, ^6^, ^7^ percutaneous stellate ganglion block,^8^, ^9^ and renal sympathetic denervation.^10^, ^11^, ^12^

Thoracic epidural anesthesia (TEA), the infusion of anesthetic agents (eg, bupivacaine or opioids) into the epidural space, is used to achieve sympathetic block at the T1 to T4 levels. TEA has the benefit of rapid onset of action and can be titrated to wanted effect. Although TEA is used primarily in the perioperative setting for pain relief, it has the potential benefit of decreasing general anesthesia requirements and postoperative arrhythmias, particularly in cardiac surgery.^13^, ^14^ TEA has been shown to increase the ventricular fibrillation threshold during acute myocardial ischemia,^15^ lengthen ventricular repolarization and effective refractory periods,^16^ suppress the effects of ventricular activation recovery interval shortening and spatial heterogeneity of repolarization caused by sympathoexcitation of the heart^17^ in animal models, and decrease arrhythmia burden in VT storm.^18^ However, its role in the short‐term management of VT storm, the selection of patients likely to benefit most, and the optimal timing of intervention are not known, limiting its adoption in clinical practice.

The purpose of the present study was to review our clinical experience with TEA in the management of VT storm and propose a systematic approach for optimal patient selection and timing of intervention. Predictors of response to TEA were also evaluated.

## Methods

### Patient Population

We reviewed data from 11 patients at the Ronald Reagan UCLA Medical Center (Los Angeles, CA) and Tsukuba University Hospital (Tsukuba, Japan), who underwent TEA for the management of VT storm between July 2005 and March 2016. Four of these patients (patients 1–4; Table 1) were included in a prior case series.^18^ Review of patient data was approved by the institutional review board at each institution, with a waiver for patient consent.

**Table 1:** Clinical Presentation

Patient	Age, y/Sex	Cardiomyopathy	LVEF, %	Time From Admission to TEA, d^a^	Clinical Scenario	VT Classification	Indication for TEA
1	75/M	ICM	30	1	Incessant VT	PMVT	Persistent VT on AAD, bridge to ablation
2	54/M	NICM	20	7	VT storm	MVT	Persistent VT on AAD, hypotension from AAD, bridge to OHT
3	66/M	HCM	60	26	VT storm	MVT	Persistent VT on AAD, ablation failed, bridge to OHT
4	75/M	NICM/sarcoid	20	11	VT storm	MVT	Persistent VT on AAD, hypotension from AAD, ablation failed
5	34/M	Cardiac lipoma	60	7	Incessant VT	MVT	Persistent VT on AAD, endocardial ablation failed, bridge to surgery
6	66/F	NICM	30	7	VT storm	PMVT	Persistent VT on AAD, hypotension and bradycardia from AAD
7	47/M	NICM	15	23	Incessant VT	MVT	Persistent VT on AAD, ablation failed
8	37/M	Brugada syndrome	50	2	Incessant VT	PMVT	Persistent VT on AAD, bradycardia on AAD
9	72/M	ICM	30	3	Incessant VT	MVT	Persistent VT on AAD, ablation failed
10	58/M	NICM	23	3	Incessant VT	MVT	Incessant runs of NSVT on AAD after unsuccessful ablation
11	62/M	ICM	30	2	Incessant VT	MVT	Persistent VT on AAD, endocardial ablation and right stellate ganglionectomy (referring center) failed, bridge to repeated ablation

AAD indicates antiarrhythmic drug; HCM, hypertrophic cardiomyopathy; ICM, ischemic cardiomyopathy; LVEF, left ventricular ejection fraction; MVT, monomorphic ventricular tachycardia; NICM, nonischemic cardiomyopathy; NSVT, nonsustained ventricular tachycardia; OHT, orthotopic heart transplant; PMVT, polymorphic ventricular tachycardia; TEA, thoracic epidural anesthesia; VT, ventricular tachycardia.

a This does not include days at outside hospitals before transfer.

### Data Collection

Baseline characteristics, VT/ventricular fibrillation burden, medical and procedural management of VT/ventricular fibrillation, and outcomes up to the time of discharge were evaluated.

Given that the effects of TEA have a rapid onset, we evaluated short‐term outcomes of TEA by comparing the number of VT episodes and shocks, whether from an implantable cardioverter defibrillator or an external defibrillator, in the 48 hours before TEA placement with that of the 48 hours after epidural anesthesia. A *complete response* was defined as complete suppression of VT episodes beginning 20 minutes after epidural anesthetic administration. *Partial response* was defined as an 80% to 99% reduction in VT episodes during this time period.^18^ Where available, we evaluated blood pressure measurements immediately before TEA initiation and 20 minutes after anesthetic administration. For patients awaiting definitive therapy (catheter ablation, CSD, or orthotopic heart transplant), we also evaluated whether interim extubation or weaning of antiarrhythmic medications was possible.

*VT storm* was defined as 3 or more sustained episodes of VT requiring intervention within a 24‐hour period, and *incessant VT* was defined as continuous sustained VT that recurred promptly despite repeated intervention for termination over several hours.^19^

### Procedure

**Figure 1:**
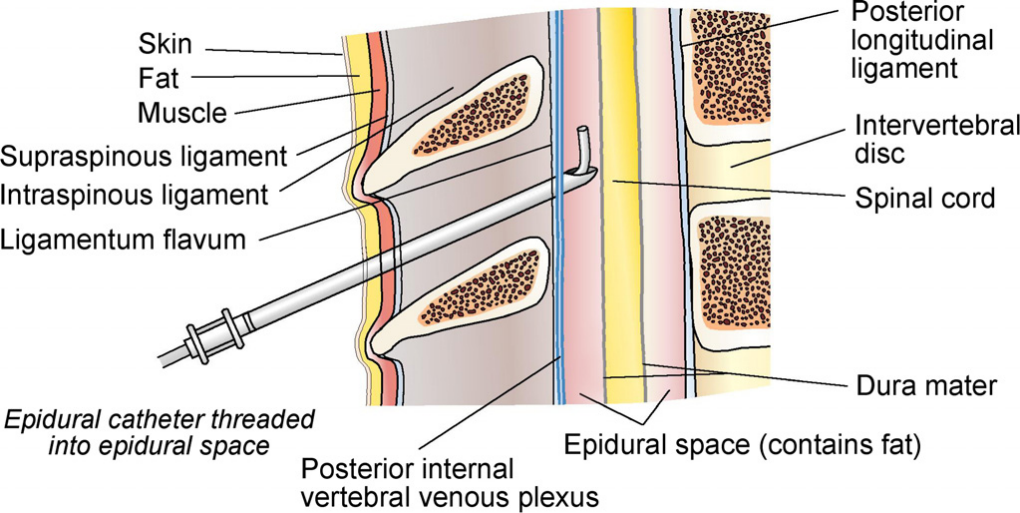
Anatomy of thoracic epidural catheter placement for the management of ventricular tachycardia storm.

The patient was placed in a right or left lateral decubitus position. Using a sterile technique, a 17‐gauge Touhy or an 18‐gauge Perican needle was inserted via a paramedian approach into the T1 to T2 or T2 to T3 interspace via a standard loss‐of‐resistance approach (Figure 1). Next, a 19‐gauge Flex‐Tip Plus or an 18‐gauge Perifix Softtip epidural catheter was advanced to 5 cm beyond the needle tip into the epidural space and secured in place. Lack of blood or cerebrospinal fluid aspiration was used to exclude intravascular or intrathecal catheter placement. In nonintubated and sedated patients, the presence of paresthesia was confirmed after injection of lidocaine. At initiation of TEA, a 1‐mL injection of bupivacaine, 0.25%, or a 10‐mL injection of ropivacaine, 0.20%, was administered via the epidural catheter, followed by an infusion at 2 mL/h of bupivacaine, 0.25%, or 3 mL/h of ropivacaine, 0.20%. The dose was titrated according to arrhythmic response.

### Statistical Analysis

The Shapiro–Wilks test was used to test for normality. Normally distributed variables were reported as mean±SD, and nonnormally distributed variables were reported as median and interquartile range (IQR). Intragroup comparisons were performed using the Wilcoxon signed‐rank test. Statistical significance was determined if *P*<0.05, with all tests being 2 tailed. Statistical analysis was performed using JMP Pro 13.

## Results

Patient characteristics and reason for initiation of TEA therapy are shown in Table 1. Of the 11 patients, 10 were male. The median age was 62 (IQR, 47–72; range, 34–75) years. Of the 11 patients, 5 (45%) had nonischemic cardiomyopathy with mean left ventricular ejection fraction of 22±6%; 3 (27%) had ischemic cardiomyopathy with mean left ventricular ejection fraction of 28±3%; and 1 (9%) each had hypertrophic cardiomyopathy, Brugada syndrome (previously undiagnosed), and cardiac lipoma, all with a normal left ventricular ejection fraction.

### Clinical Presentation and Initiation of TEA

**Figure 2:**
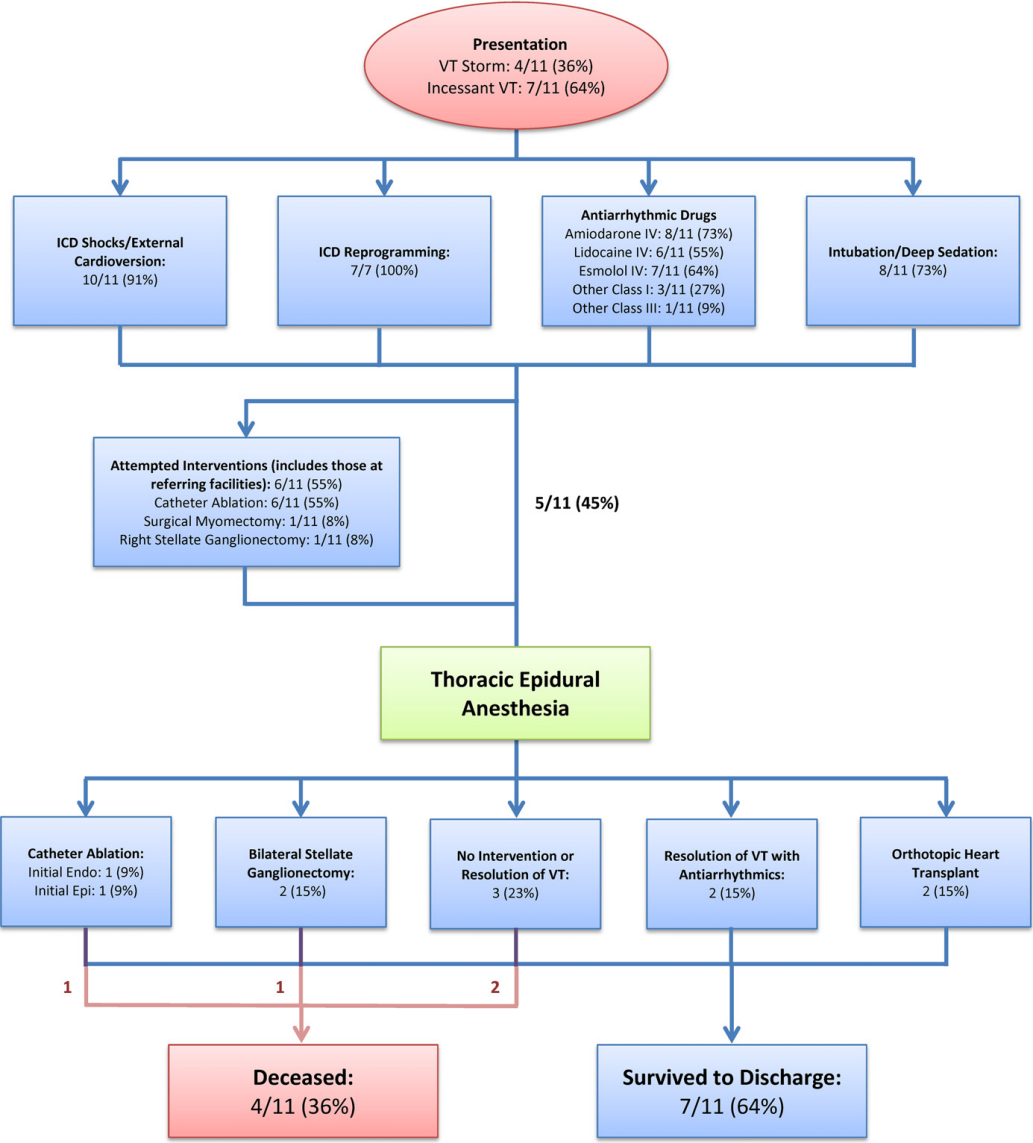
Management of ventricular tachycardia storm in study cohort. The flow chart summarizes how patients were managed before and after initiation of thoracic epidural anesthesia in our study cohort. Management with dual antiarrhythmic therapy failed in all patients, and initial interventions, both percutaneous and surgical, before initiation of thoracic epidural anesthesia, had failed in most patients. Endo indicates endocardial; Epi, epicardial; ICD, implantable cardioverter defibrillator; IV, intravenous; VT, ventricular tachycardia.

Initiation of TEA was performed at a median of 7 (IQR, 2–11; range, 1–26) days from admission or transfer, done at the discretion of the treating electrophysiologist. The clinical presentation was incessant VT in 7 patients (64%). VT morphologic features were polymorphic in 3 (27%) and monomorphic in 8 (73%). The primary indication for TEA initiation was persistent episodes of sustained VT in 10 (91%) and incessant runs of nonsustained VT in 1 (9%), despite administration of 2 or more antiarrhythmic drugs (Table 2) and correction of any electrolyte abnormalities to maintain potassium level >4.0 mmol/L and magnesium level >2.0 mEq/L. Six patients (55%) had undergone catheter ablation for VT during the same hospitalization, with early recurrence (Figure 2). TEA was initiated in 3 patients with the intention to bridge to catheter ablation (initial in 2 and repeat in the third), and orthotopic heart transplant was initiated in another 2 patients.

**Table 2:** Treatments Before TEA Initiation

Patient	AAD	Other Failed AAD	Intubated (Time Before, d)	Improvement With Deep Sedation	LV Support Device	VT Ablation During Current Hospitalization Before TEA^a^
1	Amiodarone IV, esmolol	Mexiletine	Yes (1)	No^b^		NA
2	Amiodarone IV, lidocaine	Sotalol, dofetilide	No	NA		NA
3	Amiodarone IV, lidocaine, esmolol	Sotalol	No	NA		Endo (26 d before), surgical myomectomy (15 d before)
4	Amiodarone IV, lidocaine		Yes (2)	No^b^		Epi‐Endo (2 d before)
5	Amiodarone IV, landiolol, nifekalant		Yes (1)	Yes		None
6	Procainamide IV, esmolol	Amiodarone	Yes (0)	Yes		NA
7	Amiodarone IV, lidocaine		Yes (23)	No	IABP	Endo (23 d before), Endo (7 d before)
8	Esmolol, quinidine		Yes (2)	Yes	IABP	NA
9	Amiodarone IV, lidocaine, esmolol		Yes (4)	Unable to assess^c^	IABP	Endo (1 d before)
10	Lidocaine, esmolol		No	NA		Endo (3 d before)
11	Amiodarone IV, procainamide IV, esmolol	Lidocaine	Yes (12)	Yes		Endo (13 d before, referring center)

AAD indicates antiarrhythmic drug; Endo, endocardial; Epi, epicardial; IABP, intra‐aortic balloon pump; IV, intravenous; LV, left ventricle; NA, not applicable; TEA, thoracic epidural anesthesia; VT, ventricular tachycardia.

a Includes ablations performed at referring centers before transfer if during the same hospitalization.

b Sedation limited by hypotension.

c Intubation initiated simultaneously with several other therapies.

Eight patients (73%) were intubated and deeply sedated to suppress VT at the time of TEA initiation, and 3 patients (27%) had an intra‐aortic balloon pump for hemodynamic support. Four intubated patients (50%) had documented improvement in VT burden with deep sedation (Table 2).

### Response to TEA

**Figure 3:**
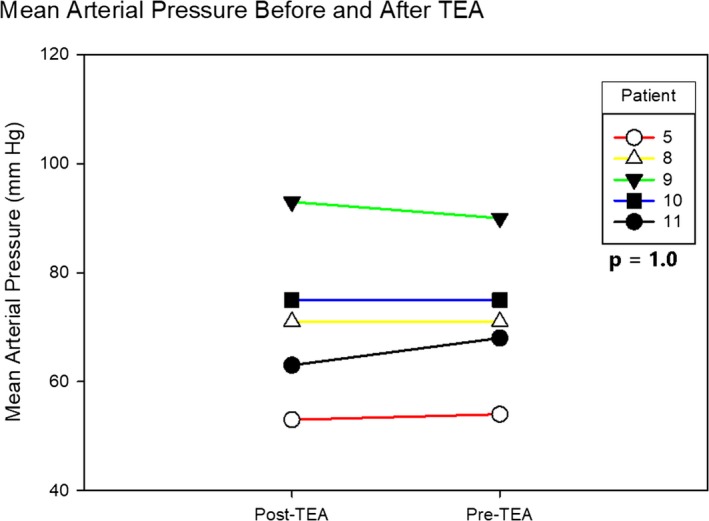
Hemodynamic response to thoracic epidural anesthesia (TEA). Mean arterial blood pressure immediately before and 20 minutes after initial TEA administration in 5 patients. No significant change in blood pressure was seen before and after TEA in these patients (Wilcoxon signed‐rank test, *P*=1.0).

**Figure 4:**
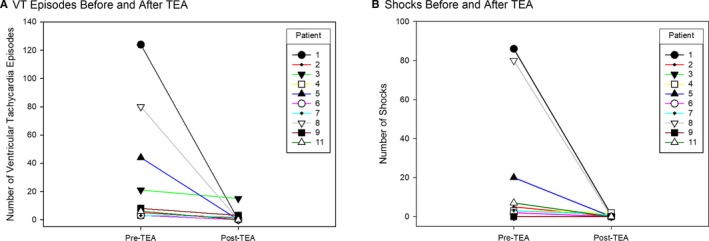
Ventricular tachycardia (VT) response to thoracic epidural anesthesia (TEA). Number of sustained VT episodes (**A**) and shocks (**B**), both external and internal, before and after initiation of TEA for each patient. Patient 10 is not included because this patient had only incessant runs of nonsustained VT.

Pre‐ and post‐TEA administration hemodynamics were available in 5 patients, with median mean arterial pressure of 71 (IQR, 61–83) mm Hg pre‐TEA and 71 (IQR, 58–83) mm Hg post‐TEA (*P*=1.0; Figure 3). Pre‐ and post‐TEA telemetry strips for measurement of electrocardiographic intervals in nonpaced rhythms were available in 3 patients. In these patients, there was no significant difference in PR, QRS duration, or QTc, with median change of 0 (IQR, −10 to 0; *P*=1.0), 0 (IQR, 0–0; *P*=1.0), and 0 (IQR, −20 to 5; *P*=1.0) milliseconds, respectively. Five patients (45%) had a complete response to TEA (patients 6, 8, and 11 had no shocks; patient 1 had a single VT episode requiring external shock 10 minutes after bupivacaine administration; and patient 5 had 2 VT episodes between 15 and 20 minutes after ropivacaine administration, which was treated with antitachycardia pacing; both had no further episodes). A sixth patient (patient 2) had a partial response with 1 episode of VT without implantable cardioverter defibrillator shock 17 hours after TEA initiation; in this patient, the bupivacaine infusion rate was increased to 3 mL/h, with no further episodes recorded (Table 3 and Figure 4). Inotropes (low‐dose dopamine, infused at a rate of <3 µg/kg per minute) were continuously required in 3 patients who responded to TEA (patients 1, 2, and 6) and 1 patient who did not respond to TEA (patient 7). Dopamine was weaned off in patients 1 and 6 as esmolol infusion was decreased after TEA initiation.

**Table 3:** Response to TEA

Patient	Anesthetic Agent Used	Maximal Continuous Infusion Rate, mL/h	Response to TEA^a^	Time Treated With TEA, d	Reason for Discontinuation	Definitive Treatment After TEA	Survived to Discharge	Reason for Death
1	Bupivacaine	2	Complete	3	Remained at time of death	Endo VT ablation (2 d later)	No	Cardiogenic/septic shock
2	Bupivacaine	3	Partial	1	Fevers, concern for catheter infection	OHT	Yes	
3	Bupivacaine	2	Nonresponder	15	Prolonged length of therapy, concern for infection risk	OHT	Yes	
4	Bupivacaine	3	Nonresponder	13	Remained at time of death	NA	No	Aspiration with asystolic arrest
5	Ropivacaine	3	Complete	8	Resolution of VT storm with AAD	Endo VT ablation (15 d later); surgical excision of cardiac lipoma (1 mo later)	Yes	
6	Bupivacaine	4	Complete	9	Prolonged length of therapy, concern for infection risk	CSD	No	Withdrawal of care per family's wishes because of lack of mental responsiveness; only brief runs of NSVT after CSD
7	Bupivacaine	2	Nonresponder	9	Resolution of VT after CSD^b^	CSD	Yes	
8	Bupivacaine	2	Complete	1	Resolution of VT storm with AAD	NA	Yes	
9	Bupivacaine	3	Nonresponder	1	Remained at time of death	NA	No	Cardiac pump failure, persistent VT, withdrawal of care
10	Bupivacaine	2	Nonresponder	4	Fevers, concern for catheter infection	NA	Yes^c^	
11	Bupivacaine	2	Complete	3	Resolution of VT after ablation	Surgical Epi VT ablation (3 d later)	Yes	

AAD indicates antiarrhythmic drug; CSD, cardiac sympathetic denervation; Endo, endocardial; Epi, epicardial; NA, not applicable; NSVT, nonsustained ventricular tachycardia; OHT, orthotopic heart transplant; TEA, thoracic epidural anesthesia; VT, ventricular tachycardia.

a Complete, no sustained VT episodes beginning 20 minutes after initiation; partial, 80% to 99% reduction in sustained VT episodes.

b Complete resolution of VT after CSD despite no response to TEA.

c Gradual resolution of incessant NSVT runs with antiarrhythmic drugs after recovery from surgery for gastroesophageal perforation.

Of the 5 complete responders, 4 (patients 1, 5, 8, and 11) had incessant VT. VT episodes and shocks decreased from a median of 65 and 50, respectively, to 0 in all of these patients after 20 minutes of therapy with TEA.

All 4 patients who responded to deep sedation had a complete response to TEA. Both patients 5 and 11 had incessant VT episodes, with any weaning of sedation before TEA; after TEA initiation, they were extubated after 48 and 12 hours, respectively.

Patients 1 and 6 were weaned off esmolol and procainamide, respectively, without recurrence of VT before undergoing VT ablation and bilateral stellate ganglionectomy, respectively.

Patient 10 (who initially had incessant VT and underwent ablation) had worsening of nonsustained VT runs after TEA, which were eventually linked to a gastroesophageal junction perforation (the cause remains unclear, but in retrospect, symptoms were noted 2 days before TEA initiation). After surgical repair, the nonsustained VT burden improved, and he was transitioned to oral antiarrhythmics.

Seven patients (64%) survived to discharge, 2 after orthotopic heart transplant. Two patients (patients 6 and 7) were bridged to CSD: patient 6 had complete suppression of VT with TEA but had VT recurrence after CSD (and discontinuation of TEA); patient 7 did not respond to TEA but had complete VT suppression after CSD and remains well 5 years later despite declining an implantable cardioverter defibrillator.

### Discontinuation of TEA

TEA was continued for a median of 4 (IQR, 2–9; range, 1–15) days. In 3 cases, the patient died, with the epidural catheter remaining in place (Table 3). Concern for catheter infection in the setting of fevers was the reason for discontinuation in 2 patients, but other sources were found in both; tip culture results were negative. Concern for infection risk because of prolonged therapy was cited for 2 patients (at 9 and 15 days after insertion). Resolution of VT was the reason for discontinuation in the other 6 patients.

### Anticoagulation Management

All anticoagulation was withheld in 6 patients (55%), including all 3 with an intra‐aortic balloon pump; prophylactic dose heparin only was continued in 4 other patients (36%). Only patient 7 had his intra‐aortic balloon pump removed while the epidural catheter remained in place, done without a prolonged weaning period. No episodes of arterial emboli were noted in any patient. Patient 1 received therapeutic anticoagulation during his endocardial VT ablation.

### Complications and Adverse Effects of TEA

No procedural complications associated with insertion or removal of the epidural catheter were identified. No epidural hematomas or infections occurred. Hypotension was noted in patient 7 when the infusion rate of bupivacaine was increased to 5 mL/h, leading to a reduction in dosage back to 4 mL/h, with resolution of hypotension.

## Discussion

Our study shows that TEA can be performed safely in patients who are seen with VT storm, and it can be effective in a subset of patients in whom initial therapies, including antiarrhythmic medications, sedation, and ablation, are not effective.

### Rationale for Use of TEA in VT Storm

The role of CSD for the management of ventricular arrhythmias in humans has been well established, exerting its effects by transiently decreasing cardiac tissue norepinephrine, increasing the threshold for ventricular arrhythmias, and reducing spatial heterogeneity in repolarization and depolarization.^3^, ^20^ Bilateral thoracic sympathectomy was first described by Estes and Izlar^4^ in 1961 to successfully treat a 35‐year‐old man with incessant runs of VT, refractory to medical therapy. Since then, CSD by surgical left or bilateral stellate ganglionectomy has been used successfully to manage recurrent ventricular arrhythmias in patients with long QT syndrome, catecholaminergic polymorphic VT, idiopathic VT, and VT associated with structural heart disease.^5^, ^21^, ^22^, ^23^, ^24^, ^25^

However, in the short‐term setting with an unstable patient, particularly a patient with incessant VT, surgical CSD may be neither feasible nor desirable. Rapidly acting and reversible methods of cardiac sympathetic blockade may be preferred in these scenarios, particularly to stop the vicious cycle of repetitive defibrillator shocks, which can cause psychological distress, potentiate heart failure, and perpetuate the heightened sympathetic state that sustains VT storm.^18^ For patients with recurrent VT after antiarrhythmic drug administration, deep sedation with intubation is the most commonly used method to reduce sympathoexcitation and avoid further arrhythmias and shocks while awaiting definitive treatment.

In comparison to TEA, deep sedation with intubation can be initiated more rapidly. However, mechanical ventilation and deep sedation can cause hypotension in patients with already compromised cardiac function, limiting the depth of sedation achievable without depending on highly arrhythmogenic inotropes. Prolonged intubation also carries risks that can contribute to morbidity/mortality.^26^, ^27^, ^28^ TEA directly blocks sympathetic neurotransmission to the heart and may be more effective than deep sedation in this regard, with potentially fewer off‐target adverse effects, such as hypotension.^29^

Hence, TEA may serve as a substitute for deep sedation. In those patients who are already intubated and are likely to require deep sedation for a prolonged period (>48 hours) before definitive therapy, TEA can serve as a bridge to allow for weaning of sedation and earlier extubation. This has the potential added benefits of allowing patients to participate in their own medical decision making and for more careful planning of definitive therapies. In some patients, TEA is sufficient to break the vicious cycle of VT storm perpetuated by sympathetic surge after shocks, allowing for effective suppression of VT with antiarrhythmics alone, before any definitive interventions are performed.

### Risks and Benefits of TEA

TEA can be initiated at the bedside or under fluoroscopy, which can help confirm catheter placement in sedated patients in whom sensory testing is not feasible. In experienced centers, the rate of complications is low: epidural hematomas occur at a rate of ≈1:20 000^30^; infections, including epidural abscess, occur at a rate of 1:1000; and meningitis occurs at a rate of 1:5000.^31^ Such complications, however, are serious, so careful selection of patients most likely to benefit from TEA is important.

### Patient Selection

VT storm represents a complex interaction of a vulnerable substrate or predisposing channelopathy and inciting triggers, including electrolyte imbalances, high sympathetic tone, triggering premature extrasystoles, and ischemia.^32^, ^33^, ^34^ Discerning which serves as the primary cause or perpetuating factor is often difficult in patients without an obvious ischemic cause. In this series, TEA responders included those with many types of cardiomyopathies and both monomorphic and polymorphic VT; most had incessant VT, suggesting a potential common factor of sympathoexcitation as the perpetuating mechanism. A decrease in VT burden with deep sedation, even if partial, suggests that sympathoexcitation plays a key role in perpetuating VT in that patient. This may explain why all such patients in our study responded completely to TEA. It is not clear, however, whether patients who do not have a decrease in VT burden with deep sedation may still respond to TEA, given the different mechanisms by which each exerts sympatholytic effects.

Weighing the potential risks and benefits, we believe that TEA should be considered if the patient: (1) has been ruled out for other inciting factors, such as acute myocardial infarction or other major noncardiac medical/surgical problem; (2) is not currently infected, is receiving dual antiplatelet therapy, or is requiring uninterrupted therapeutic anticoagulation; and (3) has incessant VT despite 2 or more antiarrhythmic medications (Table 4).

**Table 4:** Considerations for Initiation of TEA

Patient factors for which TEA could be considered
Incessant VT despite 2+ antiarrhythmic agents
Continued VT storm despite initial ablation attempt
Decrease in VT burden to deep sedation
Hypotension limiting deep sedation
Long wait time anticipated before definitive therapy
Absolute contraindications
Active infection
Dual antiplatelet therapy
Requirement for uninterrupted therapeutic anticoagulation
Relative contraindications
Acute myocardial infarction
Active major noncardiac medical or surgical process

TEA indicates thoracic epidural anesthesia; VT, ventricular tachycardia.

### Anticoagulation With TEA

Although prophylactic anticoagulation can be resumed a few hours after epidural catheter insertion or removal, therapeutic anticoagulation is not recommended while the epidural catheter is in place. Intra‐aortic balloon pump therapy should be continued at 1:1 support without heparin for the duration of TEA therapy. In patients requiring uninterrupted anticoagulation, such as those with high intracardiac thrombi burden or patients receiving extracorporeal membrane oxygenation, an alternative approach of percutaneous stellate ganglion block can be considered. However, only several case reports exist on its use in VT storm outside of the peri–myocardial infarction period.^9^, ^35^

### TEA Response as Predictor of Response to CSD

Similar to stellate ganglion block,^23^ the ability for TEA response to predict CSD response is poor: in combination with patients included in the study by Bourke et al,^18^ 2 patients had a complete response to TEA but no response to left CSD, 1 patient had no response to TEA but a complete response to bilateral CSD, and 2 patients had a compete response to TEA and had a partial or complete response to left CSD (1 each). This discrepancy in response between the 2 procedures may reflect the different degrees of autonomic modulation provided by each: sympathetic blockade with TEA occurs more centrally from that of the bilateral stellate ganglia and affects both stellate ganglia; left CSD alone may not be as effective.^5^ Sympathetic block with TEA can also be incomplete because of variability in spread of the anesthetic agent to the nerve roots or migration of the catheter; objective measurements of its sympatholytic effect are rarely performed.

### Limitations

This is a small retrospective study with evolving management of VT storm. The patients included in this study, particularly those with incessant VT, also represent some degree of selection bias given they had to be relatively hemodynamically stable, not requiring immediate extracorporeal membrane oxygenation or other ventricular assist device therapy, to undergo epidural catheter placement. In addition, this study was conducted at tertiary care centers, where patients with refractory ventricular arrhythmias are often referred. However, the insights gained from this study can be applicable to any center with expertise in TEA.

## Conclusions

TEA may be an effective therapy in the short‐term management of VT storm, especially incessant VT, refractory to initial therapies. Its minimal adverse effect profile, including hypotension, makes it a potential alternative to prolonged intubation and deep sedation in patients refractory to initial management with antiarrhythmics and/or catheter ablation. A decrease in VT burden with deep sedation suggests that sympathoexcitation plays a key role in perpetuating VT and, thus, may predict patients most likely to respond to TEA. However, patients requiring dual antiplatelet therapy or uninterrupted anticoagulation are not candidates for TEA.

## Sources of Funding

Dr. Do is supported in prat by an award from the UCLA Specialty Training and Advanced Research Program. Dr Shivkumar is supported by NIH R01 HL084261 and NIH OT2O023848.

## Disclosures

None.
